# Target trial emulation with multi-state model analysis to assess treatment effectiveness using clinical COVID-19 data

**DOI:** 10.1186/s12874-023-02001-8

**Published:** 2023-09-02

**Authors:** Oksana Martinuka, Derek Hazard, Hamid Reza Marateb, Camille Maringe, Marjan Mansourian, Manuel Rubio-Rivas, Martin Wolkewitz

**Affiliations:** 1 Institute of Medical Biometry and Statistics, Faculty of Medicine and Medical Centre, University of Freiburg, Freiburg, Germany; 2 Biomedical Engineering Research Centre (CREB), Automatic Control Department (ESAII), Universitat Politècnica de Catalunya-Barcelona Tech (UPC), Barcelona, Spain; 3 Inequalities in Cancer Outcomes Network (ICON), Department of Non-Communicable Disease Epidemiology, London School of Hygiene & Tropical Medicine, London, UK; 4 Department of Epidemiology and Biostatistics, School of Health, Isfahan University of Medical Sciences, Isfahan, Iran; 5 Department of Internal Medicine, Bellvitge University Hospital, Bellvitge Biomedical Research Institute-IDIBELL, University of Barcelona, Barcelona, Spain

**Keywords:** Bias, COVID-19, Multi-state models, Observational data, Target trial emulation

## Abstract

**Background:**

Real-world observational data are an important source of evidence on the treatment effectiveness for patients hospitalized with coronavirus disease 2019 (COVID-19). However, observational studies evaluating treatment effectiveness based on longitudinal data are often prone to methodological biases such as immortal time bias, confounding bias, and competing risks.

**Methods:**

For exemplary target trial emulation, we used a cohort of patients hospitalized with COVID-19 (*n* = 501) in a single centre. We described the methodology for evaluating the effectiveness of a single-dose treatment, emulated a trial using real-world data, and drafted a hypothetical study protocol describing the main components. To avoid immortal time and time-fixed confounding biases, we applied the clone-censor-weight technique. We set a 5-day grace period as a period of time when treatment could be initiated. We used the inverse probability of censoring weights to account for the selection bias introduced by artificial censoring. To estimate the treatment effects, we took the multi-state model approach. We considered a multi-state model with five states. The primary endpoint was defined as clinical severity status, assessed by a 5-point ordinal scale on day 30. Differences between the treatment group and standard of care treatment group were calculated using a proportional odds model and shown as odds ratios. Additionally, the weighted cause-specific hazards and transition probabilities for each treatment arm were presented.

**Results:**

Our study demonstrates that trial emulation with a multi-state model analysis is a suitable approach to address observational data limitations, evaluate treatment effects on clinically heterogeneous in-hospital death and discharge alive endpoints, and consider the intermediate state of admission to ICU. The multi-state model analysis allows us to summarize results using stacked probability plots that make it easier to interpret results.

**Conclusions:**

Extending the emulated target trial approach to multi-state model analysis complements treatment effectiveness analysis by gaining information on competing events. Combining two methodologies offers an option to address immortal time bias, confounding bias, and competing risk events. This methodological approach can provide additional insight for decision-making, particularly when data from randomized controlled trials (RCTs) are unavailable.

**Supplementary Information:**

The online version contains supplementary material available at 10.1186/s12874-023-02001-8.

## Introduction

During the coronavirus disease 2019 (COVID-19) pandemic, observational patient data have increasingly been used to evaluate treatment effectiveness, in addition to randomized controlled trials (RCTs) [1]. However, evaluating treatment effectiveness using real-world data can be challenging due to observational data limitations [2, 3]. Immortal time bias occurs when there is misalignment of start of follow-up and exposure, leading to a spurious increase in survival time for exposed patients [4]. Confounding bias relates to unequal distributions of patient’s characteristics between exposure groups, leading to an over- or underestimation of effects [5]. Furthermore, a competing risk bias occurs when competing events are treated as censoring events, and naïve Kaplan–Meier analysis is applied, leading to an overestimation of the cumulative incidence of the primary event [6, 7]. A methodological review that evaluated observational COVID-19 studies published in four high-ranking medical journals demonstrated that immortal time and confounding biases remain prevalent in pharmaco-epidemiological studies assessing treatment effectiveness when they rely on retrospective observational data [1, 2]. Ignoring the pitfalls of observational study design and the application of standard methods for survival analysis can lead to biased results and flawed conclusions [7].

The target trial emulation framework is attracting more attention, and has become a preferred method for evaluating treatment effectiveness using real-world observational data [8, 9]. During the COVID-19 pandemic, this valuable framework demonstrated its utility by providing early evidence on repurposed therapies for hospitalized patients [10, 11]. Target trial emulation can be essential to complement clinical trial findings or when RCT data are unavailable [11–14]. Importantly, this approach enables to emulate a hypothetical trial and address common observational design limitations [9]. Applying the emulated trial framework encourages researchers to carefully consider their data and setting, highlighting their strengths and limitations.

Previously, we described the extension of the target trial emulation framework to competing risk analysis, which enabled us to estimate the treatment effects on in-hospital death probabilities for COVID-19 patients, taking hospital discharge into account as a competing risk event [15]. In this article, we aim to extend the target trial emulation framework to a setting of multi-state model analysis, and demonstrate the benefits of this development on exemplary data from hospitalized patients with COVID-19. Multi-state modelling methodology enables detailed description of disease pathways in complex settings, and makes the assessment of treatment effects on heterogeneous endpoints [16, 17]. The multi-state model approach allows to account for competing events and complement the analysis of RCTs [18, 19]. Our study was motivated by the methodology used in the randomized study by Spinner et al. [20], which aimed to evaluate the effect of treatment versus standard care on the clinical status, measured on an ordinal scale, of patients with moderate COVID-19.

This paper is organized as follows: in the Method section, we first define a research question and describe the components of the emulated trial protocol; second, we describe the trial’s emulation via the clone-censor-weight technique; third, we provide a brief introduction to multi-state models and present the statistical quantities of interest. The Results section illustrates the cumulative intensities and stacked probability plots for transition probabilities, and presents odds ratios estimated using the proportional odds model. In the Discussion section, we discuss the method, alternative approaches, and limitations.

## Material and methods

Applying the emulated trial framework implies following a well-defined series of steps, we first defined the research question and hypothetical randomized trial we would have liked to conduct. Second, we drafted a study protocol with key components: eligibility and exclusion criteria, treatment strategies and assignment, a grace period, adjustment variables, time zero and end of follow-up, outcomes, and causal contrast. Third, to emulate a trial, we applied the cloning-censoring-weighting technique to our observational data [9]. Finally, we took a multi-state model approach to study disease pathways for hospitalized patients and evaluated treatment effects on different endpoints. To focus on the methodological proposal and avoid clinical interpretation issues, we excluded the pharmaceutical class of the evaluated treatment.

### Target trial emulation

#### Objective and question of clinical research

Our clinical objective was defined as follows: *to determine the effectiveness of the early administered treatment “X” compared with standard care on the clinical severity status in hospitalized patients with moderate-to-severe COVID-19.* Our clinical question of interest was: *to evaluate the effectiveness of the “X” treatment compared to the standard of care treatment (“non-X-treatment”) on clinical severity status assessed by a 5-point ordinal scale on day 30.* The key components of a hypothetical protocol are found in Supplementary Table 1. A detailed description of the emulation of the target trial and each component of the protocol is available in the article by Hernan et al. [21].

#### Protocol specification: eligibility and exclusion criteria

For this exemplary analysis, we considered hospitalized patients with moderate-to-severe COVID-19, defined as a ratio of the partial pressure of oxygen to the fraction of inspired oxygen (PaO_2_/FiO_2_) less than 300 mmHg, measured at admission [22]. We emulated the target trial using single-centre patient-level data on individuals hospitalized with COVID-19 at the Bellvitge University Hospital in Spain from March 2020 to February 2021. To meet the eligibility criteria, the patients had to be (i) 18 years of age or older and (ii) with laboratory-confirmed severe acute respiratory syndrome coronavirus type 2 (SARS-CoV-2) infection detected by polymerase chain reaction (RT-PCR), and (iii) with moderate-to-severe disease as previously defined. We excluded (i) patients with hospital-acquired COVID-19, (ii) readmitted patients, and (iii) patients who received “X” treatment before hospital admission. Additionally, we excluded from our analysis patients (*n* = 4) who had an outcome on the day of hospital admission.

#### Treatment strategies and assignment, grace period

We evaluated the effectiveness of single-dose treatment “X” together with the standard of care treatment (“X-treated”) versus the standard of care treatment alone (“non-X-treated”) according to clinical strategies. The investigated treatment is usually administered as a single dose. We defined the treatment initiation period or grace period as the five days following hospital admission, during which individuals remain eligible to both treatment arms. Exposure was defined according to the initiation of treatment within the grace period. Patients given treatment after the grace period were classified in the non-X-treated arm. The length of the grace period should be carefully defined and aligned with clinical practice [9, 23]. In this educational example, this was defined for demonstration purposes.

Based on a priori clinical knowledge, we pre-specified covariates in the protocol and included in our analysis the baseline variables of sex, age, the Charlson Comorbidity Index, and inflammation markers such as C-reactive protein (mg/L), lactate dehydrogenase (U/L), D-dimer (ng/mL) and lymphocytes (× 106/L). To account for the non-linear effect of D-dimer and reduce its skewness, we used log-transformation. The effects of other continuous biomarkers were modelled linearly. We categorized Charlson’s Comorbidity Index score as either < 2 or ≥ 2. We also included the calendar time of hospital admission, which could be potentially associated with treatment choices and changes in clinical guidelines over the course of the follow-up period. We categorized the calendar time according to the COVID-19 pandemic waves: the first wave from March through July 2020, the second wave from August through December 2020, and the third wave from January to the last available date in the dataset in February 2021.

#### Follow-up period

The start of follow-up, also called as time zero or baseline, was defined as the time of hospital admission. We pre-specified the study’s maximum follow-up time to 45 days. Patients were followed from hospital admission until either in-hospital death, discharge home, discharge to another healthcare facility (HCF), whichever occurred first, or administrative censoring at 45 days (9.2%, 46 out of 501 patients). 

#### Outcomes

The primary outcome was clinical severity status assessed on 5-point ordinal scale ranging from “discharge home” (category 1) to “in-hospital death” (category 5). We pre-specified the outcomes and ordered endpoints in five categories: 1: discharge home, 2: normal ward, 3: discharge to another HCF, 4: intensive care unit (ICU), and 5: in-hospital death. All the outcomes of interest were pre-specified and included in the protocol. We assessed the treatment effectiveness on the clinical severity status on day 30.

#### Causal contrast

In our hypothetical trial, we defined that treatment “X” could be administered within the first five days after hospital admission, in conjunction with or without other non-X treatments. Patients who deviated from the pre-specified protocol were censored. In our study, we estimated the effect similar to the per-protocol analysis. Differences between the treatment groups were determined using a proportional odds model and expressed as odds ratios.

### Practical implementation of trial with cloning, censoring, and weighting

Following the design of the target trial emulation taking the cloning-censoring-weighting approach, we created a hypothetical scenario in which a copy of each patient was assigned to both arms at hospital admission. We created two exact copies, one for the X-treated arm and one for the non-X-treated arm. Following this, the clones were censored during the grace period when they deviated from the protocol of the arm they were in. For example, patients who received treatment during the grace period were censored in the non-X-treated arm, at the time they received “X” treatment. For each patient, treatment status was defined during the 5-day grace period, and only one cloned copy of a patient was followed after this time window. Similarly, patients who never received the treatment during the grace period were censored in the treated arm at the end of the grace period. A schematic example of emulating a target trial via cloning and censoring for patients with COVID-19 is presented in Supplementary Fig. 1.

#### Estimation of the inverse probability of censoring weights

To address the selection bias introduced by artificial censoring (i.e., censoring due to cloning), we applied the inverse probability of (artificial) censoring weights. These weights correct for the censoring imposed by the study design and create a pseudo-sample in which that censoring no longer depends on the covariates. Formally, the individual unstabilized weights can be shown in Eq.  (1), and the components of the weights estimated using (2) and (3) [24, 25]. The weights are defined as the product of estimated inverse probabilities of remaining uncensored until the end of grace period conditional on the baseline (time-fixed) covariates:

1 $${\widehat{W}}_{i}\left({t}_{k}\right)=\prod_{l=1 }^{k}\frac{1}{P\left({T}_{i}^{C}>{t}_{l}\right|{T}_{i}^{C}> {t}_{l-1},{{\varvec{Z}}}_{i})}$$

where ${T}_{i}^{C}$ is time to artificial censoring with a censoring indicator ${(C}_{i}=1\space if\space censored\space \text{and}\space {C}_{i}=0\space otherwise)$ for each patient $i$ at ordered censoring times $\left(1 \le k \le 5\right)$ after the start of follow-up, where ${Z}_{i}$ are the baseline covariates. The maximum censoring time in our study was five days. The weights were computed for each arm separately.

To calculate the inverse probability of artificial censoring weight estimators, we followed the procedure available to correct for dependent censoring [25, 26]. Alternatively, the model used in the denominator of the weights can be estimated via logistic regression model [26]. Descriptions and examples of applying a logistic regression model within the target trial emulation framework, specifically focusing on the clone-censor-weight approach, can be found in the relevant studies [27, 28]. In our study, we applied semi-parametric methods [25, 29]. We first, fit censoring model to evaluate the impact of the covariates. We estimated the hazards of censoring for each arm separately conditional on the demographic and prognostic factors. We fitted a Cox regression model with the censoring indicator as a dependent variable and the time-fixed covariates as independent variables as indicated in (2):

2 $${\lambda }_{C}(t|{\varvec{Z}})={\lambda }_{0C}\left(t\right)\mathrm{exp}({\beta }_{C}{\varvec{Z}})$$

where ${\lambda }_{0C}$ is the baseline hazard, ${\beta }_{C}$ is the vector of model parameters, ${\varvec{Z}}$ represents the vector of baseline covariates. In the model, we included age, sex, the Charlson’s Comorbidity Index, C-reactive protein, lactate dehydrogenase, D-dimer, lymphocyte count, and calendar time.

Second, to estimate the probabilities of remaining uncensored for each patient $i$ on each day during the grace period, we estimated the conditional uncensored probability using the Breslow estimator (3) [29]:

3 $${\widehat{S}}_{C}\left(t|{{\varvec{Z}}}_{{\varvec{i}}}\right)=\mathrm{exp}\left\{- {\widehat{\Lambda }}_{0C}\left({t}_{j}\right)\mathrm{exp}\left({\widehat{\beta }}_{C}{{\varvec{Z}}}_{i}\right)\right\}$$

that allowed us to estimate probabilities of remaining uncensored denoted as ${\widehat{S}}_{C}\left(t|{{\varvec{Z}}}_{{\varvec{i}}}\right)$ which depend on covariates ${{\varvec{Z}}}_{{\varvec{i}}}$. Finally, we used these probabilities to calculate unstabilized weights as depicted in (1) and (4):

4 $${\widehat{W}}_{i}\left(t\right)=1/{\widehat{S}}_{C}\left(t|{{\varvec{Z}}}_{{\varvec{i}}}\right)$$

In the final step of our analysis, we included the weights in the outcome model, which we will describe in the following section.

The weights were always equal to 1 at the start of follow-up. For the X-treated arm, we obtained weights that can only change at the end of the grace period, the time at which censoring occurs. For the control arm, we obtained time-varying weights that changed within the grace period. This is due to the fact that patients in the non-X-treated arm were censored at any time during the grace period. After the grace period, the weights remained constant in both arms. As a result, copies of patients who were uncensored were up-weighted to represent the censored subjects. The mean weights were 1.8 for the X-treated arm and 1.3 for the non-X-treated arm, respectively. The weights for the non-X-treated arm were less spread. In addition, stabilization of the weights could be considered, and truncation of weights is recommended in cases of extreme weight values [24, 30]. To ensure that the weight model is correctly specified, various functional forms for the effect of continuous variables and interactions could be considered [9, 31]. We conducted a balance check to ensure that the weighting removed imbalances and that baseline covariates were well balanced between the arms [9]. We assessed balance using the standardized mean differences for each covariate in the un- and weighted sample [19]. Standardized mean differences less than 0.1 or 10% suggested good balance between the X-treated and the non-X-treated arm for each considered covariate [31].

### Multi-state model

#### Model description

**Fig. 1:**
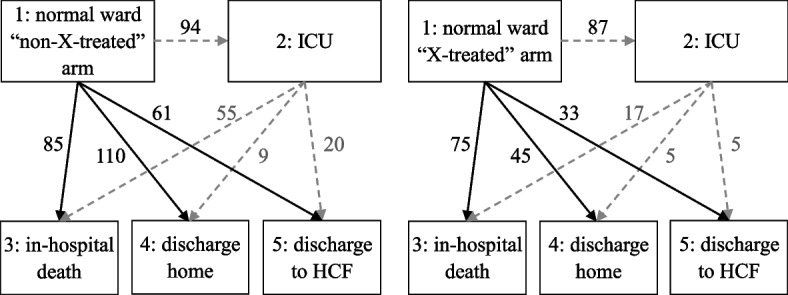
Multi-state model for COVID-19 progression. Notes: Five possible states with seven transitions and the number of patients for each transition were defined. The ICU status was modelled as an intermediate state represented in a multi-state model

We used a multi-state model to describe individual pathways across different states of hospitalized patients with COVID-19 during their follow-up time [16]. We evaluated the treatment effect using this model, comparing patients in the X-treated arm to patients in the non-X treated arm. We considered the multi-state model structure with five states (boxes): normal ward (state 1), admission to the ICU (state 2), in-hospital death (state 3), discharge home (state 4), and discharge to another HCFs (state 5) (Fig. 1). In our model, the starting state for all patients was state 1 “admitted to hospital in a normal ward”, which we considered the start of follow-up. ICU admission was modelled as an intermediate event. In our model, patients can move to three absorbing states, meaning states from which they cannot exit: in-hospital death, discharge home, or discharge to another HCFs. Possible transitions (arrows) between states were modelled and depicted in Fig. 1. For simplicity, we considered a transition model with only forward progression, that is, patients in the ICU could not go back to a normal ward.

#### Mathematical background and notations

For the multi-state analysis, we considered a non-parametric approach and quantities. We defined the transition hazards, in literature also denoted as transition intensities, as the main quantities of interest in the multi-state model framework. The transition hazards quantify the instantaneous risk of the event, that is, a transition from state *l* to state *m* at time *t.* We can write transition hazards ${\alpha }_{lm}\left(t\right)$ as it is shown in (5):

5 $${\alpha }_{lm}\left(t\right)={lim}_{\Delta t\to 0}\frac{P_{lm}\left({X}_{t+\Delta t}=m\right|{X}_{t}=l)}{\Delta t}$$

where $l \to m$ is a transition from state *l* to state *m*; ${X}_{t}$ is the state occupied at time $t$. We assumed the Markov property, which states the probability of transition to another state depends only on the present state and not on past events.

Subsequently, the cumulative transition hazards for the transition $l \to m$ until time $t$ can be defined as in (6):

6 $${A}_{lm}\left(t\right)={\int }_{0}^{t}{\alpha }_{lm}\left(u\right)du$$

All the possible transitions hazards can be collected into a matrix of transition probabilities ${\varvec{P}}(s, t)$ from state $l \to m$ within the time interval $(s, t]$ (7):

7 $${P}_{lm}\left(s, t\right)=P({X}_{t}=m|{X}_{s}=l)$$

The transition probabilities can be estimated by the corresponding matrix product-integral (8):

8 $${\varvec{P}}\left(s,t\right)=\prod_{{u}\in\left(s,t\right]}\left({\varvec{I}}+ \mathrm{d}{\varvec{A}}\left(u\right)\right)$$

where ${\varvec{I}}$ is the identity matrix and ${\varvec{A}}(t)$ is the matrix of cumulative intensities [17, 32, 33]. A detailed theoretical background and explanation of multi-state models is also found in the Handbook of Survival Analysis [34] and a practical book by Beyersmann et al. [33].

#### Data structure for multi-state analysis

For the multi-state analysis, we used the dataset and structured in a long format with one row per day for each patient. This format enabled us to account for time-varying weights. To account for the days in the rows where the event was not observed (“status = 0”), we assumed that the patient’s status was state “to = 0”. When a transition was observed, the event status changed to “1”. An example of the dataset is presented in Supplementary Table 2.

#### Statistical analysis and estimation

We proposed to estimate transition intensities and probabilities using non-parametric estimators. We defined transition intensities as in (5), and to obtain the weighted cumulative hazards we used a weighted version of the Nelson-Aalen estimator [34]. We used the individual weights calculated for each day $k$ as previously shown in (4) to the Nelson-Aalen estimator (11) where ${N}_{lm}\left(t\right)$ in (9) is the aggregated counting process and ${Y}_{l}\left(t\right)$ in (10) is the aggregated at-risk process:

9 $${N}_{lm}^{W}\left(t\right)=\sum_{i}{N}_{lmi}\left(t\right)*{\widehat{W}}_{i}\left(t\right)$$

w﻿here ${N}_{lmi}\left(t\right)\!,\,{l},\,{m} \in \mathcal{S},\,{l} \neq {m},\, {t}\leq{C}_{i}$ counting the number of direct transitions observed for subject $i$ in $\left[0,t\right]$ (${C}_{i}$ is independent right censoring)

10 $${Y}_{l}^{W}\left(t\right)=\sum_{i}{Y}_{li} \left(t\right)*{\widehat{W}}_{i}\left(t\right)$$

where $Y_{li}\left(t\right)=I\left(X_i\left(t-\right)=l\right)$ is the indicator of $X_i\left(\cdot\right)$ being in state $l$ at time $t-$. ${N}_{lm}^{W}(t)$ is the weighted number of transitions $l \to m$ up to time $t, {Y}_{l}^{W}(t)$ is the weighted number of patients at risk in state $l$ prior to time $t$ [34].

The weighted cumulative transition hazards were estimated by the weighted version the Nelson-Aalen estimator (11) [34]:

11 $${\widehat{A}}_{lm}^{W}\left(t\right)=\int_0^t\frac{\Delta{N}_{lm}^{W}\left({u}\right)}{{Y}_{l}^{W}\left({u}\right)}$$

where $$l\neq m$$.

Next, we used the weighted Aalen-Johansen estimator to calculate the weighted transition probabilities [34]. The finite matrix product obtained by including the weighted Nelson-Aalen estimator for cumulative hazards ${\widehat{A}}_{lm}^{W} \left(t\right)$ as a plug-in into the product-integral formula (8) and estimated as (12):

12 $$\widehat{{\varvec{P}}}\left(s,t\right)=\prod_{{u}\in\left(s,t\right]}({\varvec{I}}+\Delta \widehat{{\varvec{A}}}\left(u\right))$$

To conduct the multi-state model analysis in R (version 3.6.3), we used the *mstate* package. We estimated cumulative intensities via the *coxph()* function with the transition stratification *strata()* of the *survival* package. To obtain weighted cumulative hazards, the estimated *weights* were specified in the argument of the *coxph()* function. The list with elements of the unweighted hazards was replaced with the estimated baseline cumulative hazards obtained using the *basehaz()* function. Finally, the state occupation probabilities were calculated using the *probtrans()* function with the imputed weighted cumulative hazard object [32, 35]. The detailed theory and the R code applied in our analysis can be assessed in the research article by Hazard et al. [32].

Besides estimating cumulative probabilities for each outcome, we applied a weighted proportional odds model using ordinal logistic regression. We calculated a single ratio at 20, 30, and 45 days after the start of the follow-up [36]. Our outcome categories were ordered as follows: 1: discharge home, 2: normal ward, 3: discharge to another HCF, 4: ICU, and 5: in-hospital death. For the 45-day follow-up, we used the same categories except the normal ward category. By ordering outcome categories from best to worst, an odds ratio greater than 1 indicates a difference in clinical status distribution towards severity for the X-treated arm compared to the non-X-treated arm [20]. Confidence intervals (CIs) (95%) were calculated using non-parametric bootstrapping with 1000 replicates. We also conducted sensitivity analysis by imputing missing values for the covariates, assuming that data were missing at random. The description of the cohort and the number of transitions in the multi-state model with imputed data are provided in Supplementary Table 3 and Supplementary Fig. 2.

## Results

### Data overview

In total, 501 (83.6%) individuals with complete records met our eligibility criteria and were qualified for emulation. A total of 124 (24.8%) patients were treated with treatment “X” within five days of hospital admission. Only 20 (4%) patients were treated after the grace period and assigned to the non-X-treated arm. Demographic and prognostic characteristics prior to cloning are found in Supplementary Table 4. All 501 patients were cloned, with each clone entering the X-treatment and non-X-treatment arm. Supplementary Fig. 3 illustrates the absolute standardized differences before and after applying the weights. None of the standardized mean differences in the weighted sample exceed the cut-off of 0.1 (Supplementary Fig. 3). We demonstrated that the patient’s demographic and prognostic characteristics were well balanced after applying the weights (Supplementary Fig. 3).

### Cumulative hazards and predicted probabilities

**Fig. 2:**
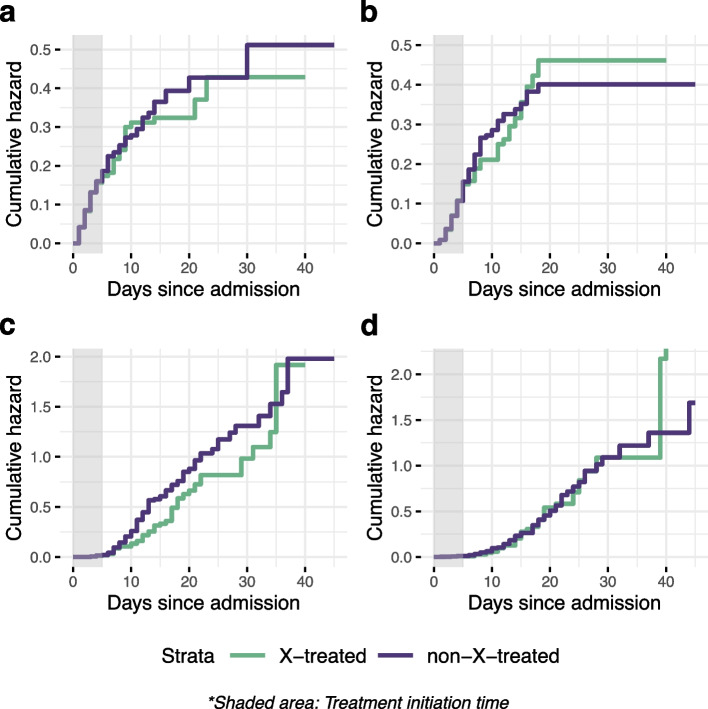
Weighted estimated cause-specific cumulative hazards from the normal hospital ward using the Nelson-Aalen estimator. **a-d** Illustrates transitions from normal ward. **a** From normal ward to ICU. **b** From normal ward to in-hospital death. **c** From normal ward to discharge home. **d** From normal ward to discharge to another HCF

We obtained cumulative hazards and predicted probabilities from our emulated trial. The estimated and visualized weighted cause-specific cumulative hazards starting from the normal hospital ward for the X-treated and non-X-treated arm, are shown in Fig. 2. None of these cumulative hazards revealed evidence of significant differences between the two arms in the investigated outcomes.

**Fig. 3:**
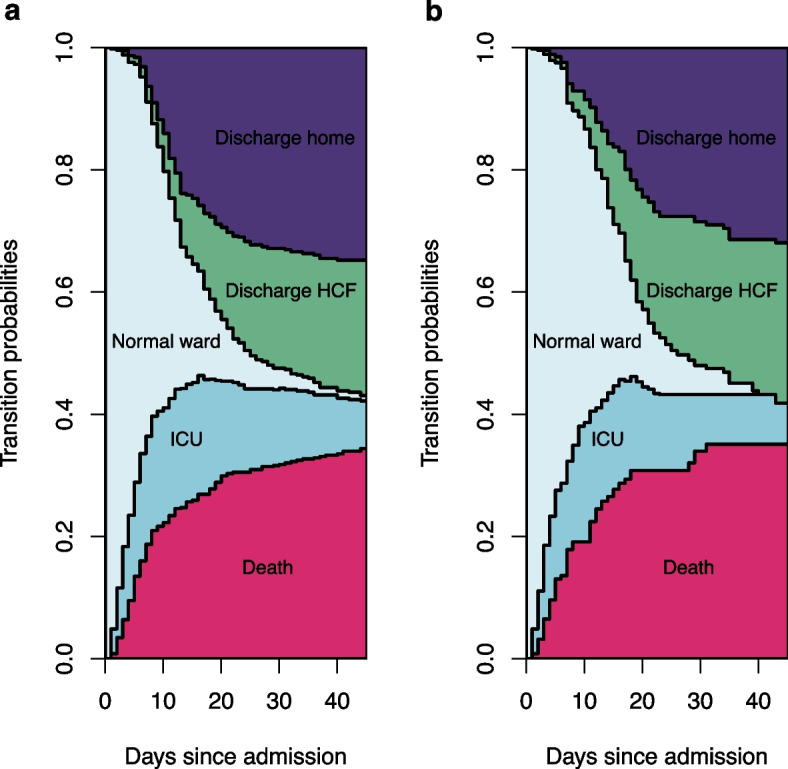
Weighted results for transition rates starting from hospital admission. Notes: **a** Non-X-treated arm. **b** X-treated arm. State 1: Normal ward. State 2: Admission to ICU. State 3: In-hospital death; State 4: Discharge home. State 5: Discharge to another HCF

The transition rates of the outcomes we investigated were similar between the two treatment arms (Fig. 3). At day 30, the predicted probability of in-hospital death outcome were 34.0% for the X-treated arm and 31.6% for the non-X-treated arm. The probabilities of ICU stay were 8.1% for the X-treated and 12.4% for the non X-treated. The probabilities of discharge home were 28.5% for the X-treated and 32.9% for the non-X-treated. The probabilities of being discharge to another HCF were 23.5% for the X-treated and 19.6% for the non-X-treated. At the last day of the follow up, the transition rates for in-hospital death were 35.1% in the X-treated and 34.4% in the non-X-treated. The probabilities of ICU stay were 6.7% for the X-treated and 7.8% for the non X-treated arm. The probabilities of being discharged home were 31.9% in the X-treated and 34.8% in the non-X-treated. The probabilities of being discharged to another HCF were 26.2% in the X-treated and 22.2% in the non-X-treated.

The proportional odds ratio at 30 days was 1.11 (95% CI: 0.82–1.51). Additionally, we calculated the proportional odds ratio at 20 and 45 days, which indicated a negative X treatment effect with a weighted odds ratio, 1.09 (95% CI: 0.78–1.52) and 1.08 (95% CI: 0.79–1.50), respectively. The results of the completed case analysis (n = 599) with imputed missing values for the covariates are provided in the Supplementary Figs. 4–6. This data analysis yielded results consistent with the primary analysis (Supplementary Figs. 5–6); the proportional odds at 20, 30, and 45 days were 1.02 (95% CI: 0.77–1.39), 1.07 (95% CI: 0.80–1.45), and 1.04 (95% CI: 0.78–1.40) respectively.

## Discussion

This methodological study provides a demonstration of the target trial emulation framework, with an extension to multi-state analysis. In this study, we carried out a target trial emulation taking the clone-censor-weight approach that avoids immortal time bias and confounding bias. Specifically, we focussed on addressing competing risks and extended standard survival analysis to the multi-state model. This combined approach allowed us to demonstrate the common biases and revealed a solution to facilitate the evaluation of treatment effectiveness by considering clinically meaningful time-to-event outcomes. We applied the methodology in the context of assessing of the effectiveness of a single-dose, time-dependent treatment using routinely collected observational hospital data of patients with COVID-19. As this is a methodological demonstration of the application of the target trial emulation in settings in which competing events occur and are of interest, we did not interpret our emulated study’s results from the perspective of clinical importance.

To mitigate immortal time in our emulated study, we defined the eligibility criteria, the time zero of follow-up, and the grace period for treatment initiation and applied the clone-censor techniques. Time-fixed confounding bias was addressed by cloning; as such, each patient’s copy had the same values of the baseline covariates. The inverse probability weighting was then used to correct for censoring imposed by the study design. Alternative to the clone-censor-weight approach with a grace period, the sequential target trial within the framework of target trial emulation can be used [8, 37]. The main difference between these two methods is that the sequential trial approach incorporates a sequence of nested trials where each new trial starts for patients not yet under the treatment of interest, at successive times. Instead of the cloning-censoring-weighting method requiring that all patients enter both arms with future censoring at deviation from the protocol. Both methods allow to account for immortal time bias [37].

Competing risks are an important issue that can occur in the analysis of survival data of both RCTs and observational studies [19]. In the presence of competing events, several approaches are available for estimating the treatment effect, depending on the causal question of interest. These approaches can include the estimation of effects on the composite outcome, total effects, or direct effects [9, 38, 39]. In the case of estimation of total effect, the treatment effect on the primary outcome is determined by taking into account the treatment effects on the competing events. This implies that the treatment effect on the primary outcome includes all the paths “mediated” through the competing events [38, 39]. Although the total effect need to be interpreted with caution, we argue that this approach might be more important when assessing treatment effectiveness for hospitalized patients with COVID-19. This is because it allows obtain additional information on clinically important opposite endpoints and might be essential for various decision-makers and for the clinical management of COVID-19 [16, 32, 40, 41]. Another option is the estimation of the direct effect, which is more suitable when a causal question on treatment mechanisms is exclusively investigated [39]. The direct effect quantifies that the effect of treatment that is not “mediated” by the competing events and implies a hypothetical situation where competing events are eliminated by considering them as censored events [39]. A detailed description of the choice of statistical methods in a setting with competing events and estimating total or direct effect is found in the research article by Young et al. [38].

In our study, we used the multi-state model and examined the treatment effects on clinical severity status by accounting for all pathways, that is, the intermediate and competing events. This allowed us to achieve a comprehensive and informative assessment, similarly to the motivating study by Spinner et al. [20]. Previously, von Cube et al. emphasized that in RCTs entailing comprehensive treatment evaluation, it is important to assess treatment effects on all clinically meaningful endpoints [16]. In our study, we followed patients with moderate to severe COVID-19 from the hospital’s normal ward which is a more controlled environment. We considered ICU admission as an intermediate state, but our trial could also be emulated from a new time zero for severe patients requiring intensive care. In addition, this would require baseline data on prognostic covariates to permit future comparison between the two arms [14]. We also separated two discharge-alive reasons: discharge home and discharge to another HCF. Patients discharged to another HCF could potentially carry a higher risk of death than patients discharged home. Furthermore, it’s also possible to distinguish between discharge from normal ward to another HCF and discharge from ICU to another HCF.

There are several general principles that should be followed to emulate the target trial successfully, and we want to emphasize some of them here. First, emulating a target trial requires the use of extensive data from multicentre registries to provide reliable results and ensure generalizability [21]. Second and foremost, the causal clinical question of interest must be well-defined and the study protocol, together with its components, must be specified. The emulated target trial’s protocol must include all key elements described by Hernan et al. [21]. A grace period should be discussed with clinicians and determined by clinical relevance [23]. For COVID-19 patients, this period should be short enough to avoid heterogeneity between treatment arms. Also, it is essential to include all clinically important factors and strongly prognostic covariates [19]. For this purpose, directed acyclic graphs can be used to identify causal relationships among variables and reduce confounding bias [40]. In the presence of competing risks, as outlined by Rojas-Saunero et al., the question of clinical interest leading to estimation of the total or direct effect should be first defined first before choosing the statistical analysis method [39]. Finally, to assess the validity of findings, data from emulated trials can be compared with those obtained from RCTs or other meta-analyses. Future potential methodological and clinical extensions of our framework include assessing dynamic treatment strategies by including time-dependent prognostic covariates, and applying G-methods; identifying the three assumptions of positivity, consistency, and exchangeability that are required to estimate causal treatment effects [30], and considering additional states in a multi-state model, such as respiratory support.

## Limitations

Our study has important limitations associated with the demonstrative and methodological concept of our emulated trial. First, we emulated the target trial using a small sample from a single-centre. Second, we chose a 5-day grace period for illustrative purposes, whereas in clinical studies the length of a grace period should be defined according to clinical relevance [9, 23]. Third, for demonstrative purposes, we included only baseline covariates, but post-baseline time-varying covariates of clinical severity could be considered. However, the effects of time-dependent covariates should be interpreted with caution and require appropriate method use [41]. Additionally, in our study, vaccination status could be included as a covariate because the vaccination-induced immunological status is associated with a significant decrease in patients with severe illness and mortality in COVID-19 [42]. Fourth, standard of care has evolved during the pandemic entailing various treatments and combination therapies for patients with COVID-19. Based on all these shortcomings, our emulated hypothetical trial and protocol can differ considerably from a pragmatic trial.

In our study, we also acknowledge certain limitations related to statistical analysis. First, in multi-state analysis, we rely on the Markov assumption, defined as the probability of moving to the next state depends only on the actual state. Second, we excluded patients with missing data in the prognostic covariates but considered imputation in the additional analysis. It is advised to consider limitations related to missing data in a target trial and possible approaches, especially when data is incomplete on the variables determining eligibility criteria [43]. Finally, we summarised the treatment effect applying the odds ratio from the proportional odds model involving ordering the outcomes, which enabled a simple interpretation about the treatment effect on clinical severity [20, 44]. However, this summary parameter requires proportional odds assumption, and entails the principal limitation of interpretation [36]. Therefore, alternative target parameters, which allow for a better understanding and interpretation of results such as rates, ratios, and restricted length of stay in each state, could be presented [45].

## Conclusions

In summary, this target trial emulation approach with extended multi-state model analysis enables treatment effectiveness to be evaluated on clinically important endpoints making the best use of real-world observational data. This combined methodological approach address many of the common limitations of observational data, providing an evaluation of treatment effectiveness, and enhancing our understanding of the clinical course of patients with COVID-19. Combining two state-of-the-art methodologies helps avoid immortal time bias, confounding bias, and competing events and to obtain unbiased evidence from observational data. Our framework could be extended to evaluate the effectiveness of time-varying treatments and to consider additional clinical states in the multi-state model.

### Supplementary Information

**Additional file 1: Table 1. **A summary of protocol components for the target trial emulation. **Table 2. **Example of the structure of the data set used for multi-state analysis in the non-X-treated arm. **Table 3.** Baseline characteristics for eligible patients according to X-treatment receive within 5-days before cloning and censoring, complete dataset with missing value imputation, *n*=599. **Table 4.** Baseline characteristics for eligible patients according to categorization of 5-day grace period before cloning. **Fig. 1.** Example of target trial emulation with cloning and censoring for COVID-19 patients. **Fig. 2.** Multi-state model for COVID-19 progression, complete dataset with missing value imputation, *n*=599. **Fig. 3.** Covariate balance using standardised mean differences at five day grace period before and after applying inverse probability of artificial censoring weighting. **Fig. 4.** Covariate balance using standardised mean differences at five day grace period before and after applying inverse probability of artificial censoring weighting, complete dataset with missing value imputation, *n*=599. **Fig. 5. **Weighted estimated cause-specific cumulative hazards from the normal hospital ward using the Nelson-Aalen estimator, complete dataset with missing value imputation, *n*=599. **Fig. 6.** Weighted results for transition rates starting from hospital admission, complete dataset with missing value imputation, *n*=599.

## Data Availability

The code for trial emulation was adapted according to the Maringe et al. tutorial [9]. The extended code of this study are available from the corresponding author. Data for this exemplary study are considered sensitive and are not publicly available. The point of contact for queries about the data and for data review is O. Martinuka.

## Abbreviations

*CI*: Confidence interval
*COVID-19*: Coronavirus disease 2019
*ICU*: Intensive care unit
*HCF*: Healthcare facility
*RCTs*: Randomized controlled trials
*PaO*_*2*_*/FiO*_*2*_: Partial pressure of oxygen in arterial blood (PaO_2_) to the fraction of inspiratory oxygen concentration (FiO_2_)
