# Supplementary material for: Target trial emulation with multi-state model analysis to assess treatment effectiveness using clinical COVID-19 data
Source: BMC Med Res Methodol. 2023 Sep 2;23:197. doi: 10.1186/s12874-023-02001-8 (PMC10474639; doi:10.1186/s12874-023-02001-8)
Supplement: Supplementary file 1 — Additional file 1: Table 1. A summary of protocol components for the target trial emulation. Table 2. Example of the structure of the data set used for multi-state analysis in the non-X-treated arm. Table 3. Baseline characteristics for eligible patients according to X-treatment receive within 5-days before cloning and censoring, complete dataset with missing value imputation, n=599. Table 4. Baseline characteristics for eligible patients according to categorization of 5-day grace period before cloning. Fig. 1. Example of target trial emulation with cloning and censoring for COVID-19 patients. Fig. 2. Multi-state model for COVID-19 progression, complete dataset with missing value imputation, n=599. Fig. 3. Covariate balance using standardised mean differences at five day grace period before and after applying inverse probability of artificial censoring weighting. Fig. 4. Covariate balance using standardised mean differences at five day grace period before and after applying inverse probability of artificial censoring weighting, complete dataset with missing value imputation, n=599. Fig. 5. Weighted estimated cause-specific cumulative hazards from the normal hospital ward using the Nelson-Aalen estimator, complete dataset with missing value imputation, n=599. Fig. 6. Weighted results for transition rates starting from hospital admission, complete dataset with missing value imputation, n=599. [file 12874_2023_2001_MOESM1_ESM.docx]

**Supplementary Information**

**BMC Medical Research Methodology**

**Target trial emulation with multi-state model analysis to assess treatment effectiveness using clinical COVID-19 data**

Oksana Martinuka, Derek Hazard, Hamid Reza Marateb, Camille Maringe, Marjan Mansourian, Manuel Rubio-Rivas, Martin Wolkewitz

**Supplementary Table 1.** A summary of protocol components for the target trial emulation

**Supplementary Table 2.** Example of the structure of the data set used for multi-state analysis in the non-X-treated arm

**Supplementary Table 3.** Baseline characteristics for eligible patients according to X-treatment receive within 5-days before cloning and censoring, complete dataset with missing value imputation, *n=599*

**Supplementary Table 4.** Baseline characteristics for eligible patients according to categorization of
5-day grace period before cloning

**Supplementary Fig. 1.** Example of target trial emulation with cloning and censoring for COVID-19 patients

**Supplementary Fig. 2.** Multi-state model for COVID-19 progression, complete dataset with missing value imputation, *n=599*

**Supplementary Fig. 3.** Covariate balance using standardised mean differences at five day grace period before and after applying inverse probability of artificial censoring weighting

**Supplementary Fig. 4.** Covariate balance using standardised mean differences at five day grace period before and after applying inverse probability of artificial censoring weighting, complete dataset with missing value imputation, *n=599*

**Supplementary Fig. 5.** Weighted estimated cause-specific cumulative hazards from the normal hospital ward using the Nelson-Aalen estimator, complete dataset with missing value imputation, n=599

**Supplementary Fig. 6.** Weighted results for transition rates starting from hospital admission, complete dataset with missing value imputation, *n=599*

**Supplementary Table 1.** A summary of protocol components for the target trial emulation

| **Protocol component** | **Description of emulation** |
| --- | --- |
| Clinical question | To evaluate the effectiveness of the “X” treatment compared to the standard of care treatment (“non-X-treatment”) alone on clinical severity status assessed by a 5-point ordinal scale on day 30 |
| Eligibility criteria | - moderate-to-severe patients with PaO2/FiO2 ratio less than 300 mmHg - adult patients aged ≥18 years - PCR confirmed SARS-CoV-2 infection |
| Exclusions | - hospital-acquired COVID-19 - patients who were readmitted - patients who received treatment before hospital admission |
| Treatment strategies | - “X” single dose treatment with standard of care treatment (“X-treated”) versus the standard of care treatment alone (“non-X-treated”) - Initiate treatment (“X-treated”) within five days of hospital admission - Do not initiate treatment (“non-X-treated”) within five days of hospital admission |
| Treatment assignment | Non-randomized treatment assignment |
| Grace period | First five days after hospital admission |
| Adjustment variables | - Demographic: age and sex - Comorbidities: Charlson Comorbidity Index - Clinical prognostic biomarkers: C-reactive protein, lactate dehydrogenase, D-dimer, lymphocyte count - Calendar time: pandemic waves with three categories |
| Follow-up time | - Begins with hospital admission, and treatment initiation must occur within the first five days after hospitalization and ends with in-hospital death, discharge home, discharge to other health care facility or administrative censoring at 45 days - We assessed the clinical severity status on day 30 |
| Outcomes | - Primary outcome: clinical severity status on a 5-point ordinal scale - Consisting of the following categories of endpoints: 1: discharge home, 2: normal ward, 3: discharge to another HCF; 4: ICU; and 5: in-hospital death |
| Contrast of interest | Observational analogue of the per-protocol effect |
| Analysis plan | - Emulation of target trial: Analysis conducted on the cloned data set reproducing random treatment assignment and with a censoring indicator when there is a protocol deviation within the grace period. Informative censoring (due to cloning and censoring) accounted for by using inverse probability of artificial censoring weights. - Multi-state model analysis |
| **Abbreviations:** COVID-19, coronavirus disease 2019; HCF, healthcare facility; ICU, intensive care unit; PaO_2_/FiO_2_, oxygen in arterial blood (PaO_2_) to the fraction of inspiratory oxygen concentration (FiO_2_); PCR, polymerase chain reaction; SARS-CoV-2, severe acute respiratory syndrome coronavirus type 2 | |

**Supplementary Table 2.** Example of the structure of the data set used for multi-state analysis in the non-X-treated arm (time in days)

| **ID** | **States** | | **Times** | | **Status** | **Weight** |
| --- | --- | --- | --- | --- | --- | --- |
|  | **from** | **to** | **entry** | **exit** |  |  |
| 1 | 1 | 0 | 1 | 2 | 0 | 1.00 |
| 1 | 1 | 0 | 2 | 3 | 0 | 1.05 |
| 1 | 1 | 0 | 3 | 4 | 0 | 1.05 |
| 1 | 1 | 0 | 4 | 5 | 0 | 1.09 |
| 1 | 1 | 0 | 5 | 6 | 0 | 1.09 |
| … | … | … | … | … | … | … |
| 1 | 1 | 4 | 9 | 10 | 1 | 1.09 |

**Supplementary Table 3.** Baseline characteristics for eligible patients according to X-treatment receive within 5-days before cloning and censoring, complete dataset with missing value imputation^*^, *n=599*

|  | **X-treated group (N=141) ^b^** | **Non-X-treated group ^a^**  **(N=458) ^b^** | **Overall (N=599)** |
| --- | --- | --- | --- |
| **Sex** |  |  |  |
| Male | 108 (76.6%) | 316 (69.0%) | 424 (70.8%) |
| Female | 33 (23.4%) | 142 (31.0%) | 175 (29.2%) |
| **Age** |  |  |  |
| Median [IQR] | 67.2 [57.6, 74.1] | 69.9 [59.9, 78.2] | 70 [59.5, 77.2] |
| Mean (SD) | 65.8 (12.3) | 68.8 (13.1) | 68.1 (13.0) |
| **Charlson Comorbidity Index** |  |  |  |
| <2 | 115 (81.6%) | 321 (70.1%) | 436 (72.8%) |
| ≥2 | 26 (18.4%) | 137 (29.9%) | 163 (27.2%) |
| **C-reactive protein, mg/L** |  |  |  |
| Median [IQR] | 147 [90, 241] | 121 [70, 193] | 126 [74, 206] |
| Mean (SD) | 168 (104) | 147 (108) | 152 (107) |
| **Lactate dehydrogenase, U/L** |  |  |  |
| Median [IQR] | 415 [326, 502] | 400 [303, 524] | 404 [308, 517] |
| Mean (SD) | 448 (190) | 431 (185) | 435 (186) |
| **D-dimer, ng/mL** |  |  |  |
| Median [IQR] | 508 [304, 1 016] | 423 [274, 915] | 438 [279, 949] |
| Mean (SD) | 1 529 (5 003) | 2 069 (1 055) | 1 942 (9 539) |
| **Lymphocytes**, ×106/L |  |  |  |
| Median [IQR] | 800 [550, 1 070] | 795 [540, 1 108] | 800 [540, 1 105] |
| Mean (SD) | 859 (475) | 863 (450) | 862 (455) |
| **Calendar time ^c^** |  |  |  |
| First wave | 92 (65.2%) | 272 (59.4%) | 364 (60.8%) |
| Second wave | 27 (19.1%) | 100 (21.8%) | 127 (21.2%) |
| Third wave | 22 (15.6%) | 86 (18.8%) | 108 (18.0%) |

**Abbreviations:** IQR, interquartile range; SD, standard deviation

**Notes:**

^a^ non-X-treated-group: standard of care treatment

^b^ we considered patients treated with “X” vs. those not treated with “X” within a five-day period, including early in-hospital deaths in that group, using the dataset before cloning and censoring

^c^ first wave: from March to July 2020; Second wave: from August to December 2020; Third wave: from January 2021 to March 2021

**^*^ Description of imputation**

We performed a single imputation for missing values of the prognostic covariates, assuming that they were missing at random. This imputation was performed using the *mice* (Multivariate Imputation by Chained Equations) package in R.

**Supplementary Table 4.** Baseline characteristics for eligible patients according to X-treatment receive within 5-days before cloning and censoring

|  | **X-treated group (N=124) ^b^** | **Non-X-treated group ^a^**  **(N=377) ^b^** | **Overall (N=501)** |
| --- | --- | --- | --- |
| **Sex** |  |  |  |
| Male | 92 (74.2%) | 257 (68.2%) | 349 (69.7%) |
| Female | 32 (25.8%) | 120 (31.8%) | 152 (30.3%) |
| **Age** |  |  |  |
| Median [IQR] | 67 [58, 74] | 70 [68, 78] | 70 [60, 78] |
| Mean (SD) | 66.5 (12.3) | 69.4 (13.1) | 68.6 (12.9) |
| **Charlson Comorbidity Index** |  |  |  |
| <2 | 101 (81.5%) | 261 (69.2%) | 362 (72.3%) |
| ≥2 | 23 (18.5%) | 116 (30.8%) | 139 (27.7%) |
| **C-reactive protein, mg/L** |  |  |  |
| Median [IQR] | 149 [88, 241] | 123 [70, 193] | 131.2 [74.3, 209.7] |
| Mean (SD) | 171 (106) | 147 (108) | 153 (108) |
| Missing **^c^** | 1 (0.6%) | 3 (0.7%) | 4 (0.7%) |
| **Lactate dehydrogenase, U/L** |  |  |  |
| Median [IQR] | 416 [326, 509] | 399 [305, 525] | 405 [312, 518] |
| Mean (SD) | 449 (178) | 434 (189) | 438 (186) |
| Missing **^c^** | 14 (8.5%) | 56 (12.9%) | 70 (11.7%) |
| **D-dimer, ng/mL** |  |  |  |
| Median [IQR] | 466 [278, 964] | 401 [266, 904] | 411.0 [267, 913] |
| Mean (SD) | 1 560 (5 318) | 1 765 (5 196) | 1 714 (5 222) |
| Missing **^c^** | 8 (4.8%) | 34 (7.8%) | 42 (7.0%) |
| **Lymphocytes**, ×106/L |  |  |  |
| Median [IQR] | 765 [520, 1 060] | 790 [530, 1 100] | 790 [530, 1 100] |
| Mean (SD) | 839 (489) | 894 (652) | 880 (616) |
| Missing **^c^** | 2 (1.2%) | 10 (2.3%) | 12 (2.0%) |
| **Calendar time ^d^** |  |  |  |
| First wave | 83 (57.6%) | 205 (57.4%) | 288 (57.5%) |
| Second wave | 19 (13.2%) | 92 (25.8%) | 111 (22.2%) |
| Third wave | 22 (15.3%) | 80 (22.4%) | 102 (20.4%) |

**Abbreviations:** IQR, interquartile range; SD, standard deviation

**Notes:**

^a^ non-X-treated-group: standard of care treatment

^b^ we considered patients treated with “X” vs. those not treated with “X” within a five-day period, including early in-hospital deaths in that group, using the dataset before cloning and censoring

^c^ missing category: we calculated the missing number and proportion from the cohort (n = 599) before excluding the missing data

^d^ first wave: from March to July 2020; Second wave: from August to December 2020; Third wave: from January 2021 to March 2021

**Supplementary Fig. 1.** Example of target trial emulation with cloning and censoring for COVID-19 patients


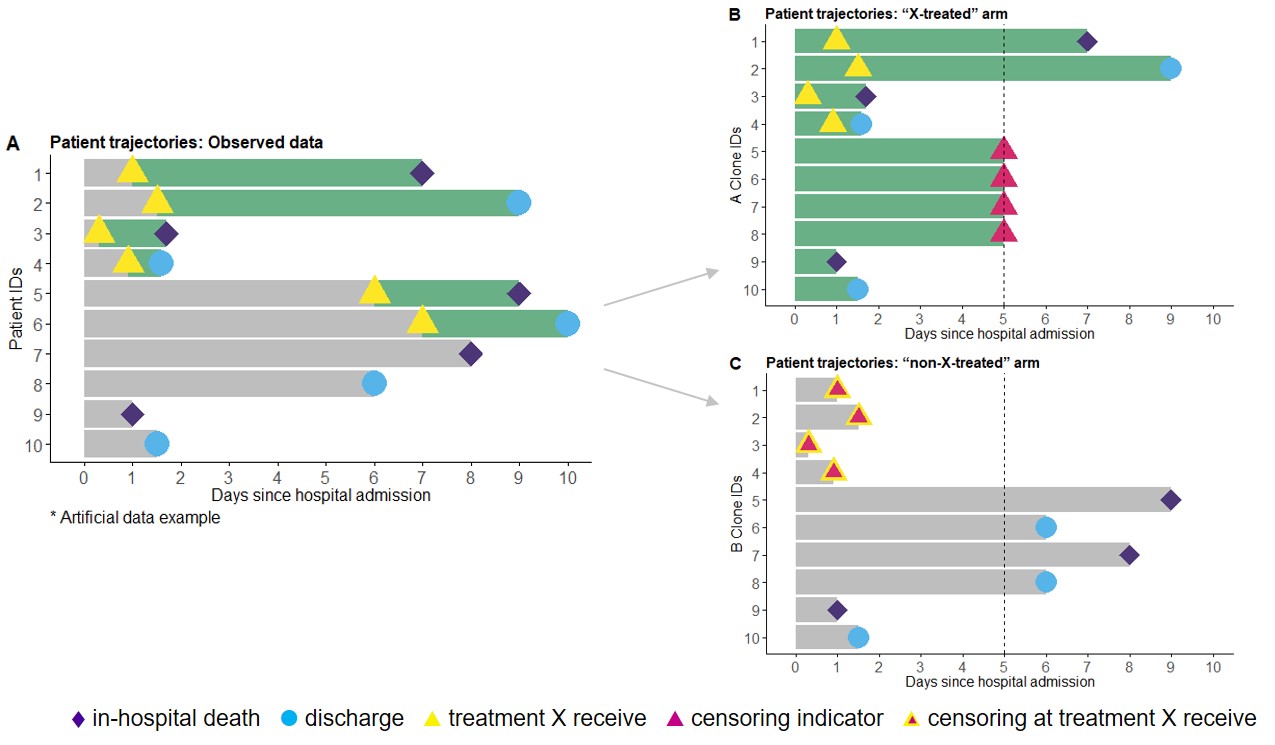


**Notes:** In figure (A) on the left is an example of initial observational hospital data: Patients with identification (ID) numbers 1 to 6 received treatment “X”. Patients with ID 7-10 never received treatment “X” during their hospital stay. Patients with ID 1, 3, 5, 7, and 9 died and 2, 4, 6, 8, and 10 were discharged from hospital (to home or to another healthcare facilities).

By emulating, two copies of the observed data are created for each patient, with one clone assigned to the intervention arm (X-treated) and another to the control arm (non-X-treated), thus increasing the sample size twice:

- The X-treated arm (figure B): patients were censored when they deviated from the planned protocol; these are individuals with IDs 5 to 8 who were never treated or treated later (with “X”) after the grace period and were censored at the end of the 5-day grace period.
- The non-X-treated arm (figure C): patients were censored when they deviated from the planned protocol at the time of treatment (“X”) initiation; these are individuals with IDs 1–4 who received “X” treatment within the grace period.
- In both arms (figures B and C): patients who were untreated and experienced an outcome within the grace period (IDs 9 and 10) are considered.

**Supplementary Fig. 2.** Multi-state model for COVID-19 progression, complete dataset with missing value imputation, *n=599*

**
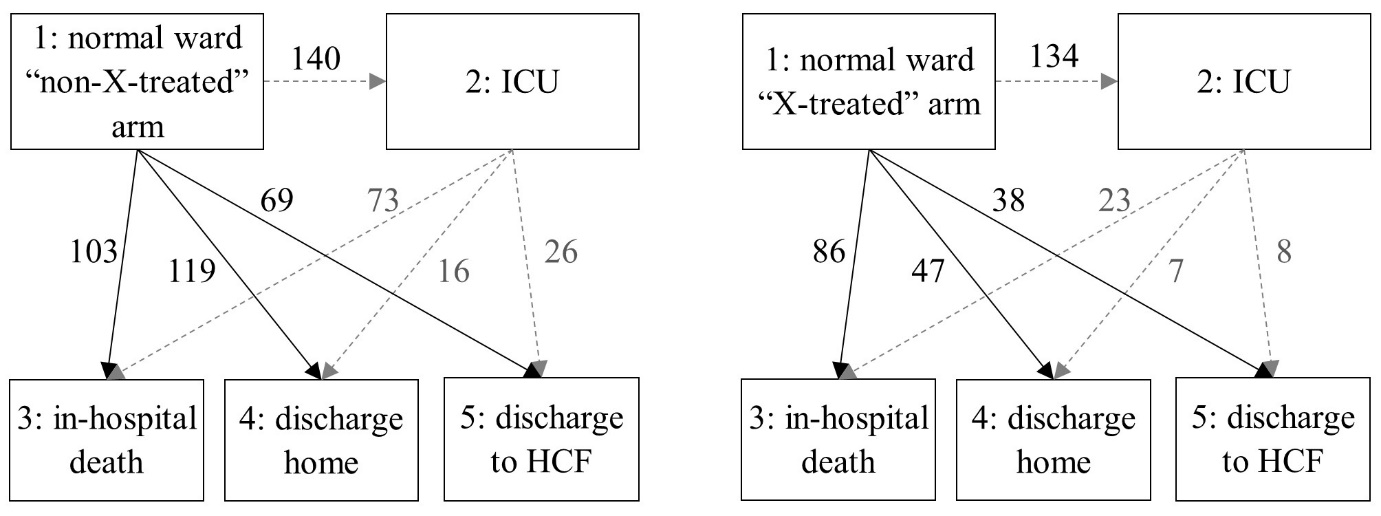
**

**Notes:** Five possible states with seven transitions and the number of patients for each transition were defined. The ICU status was modelled as an intermediate state represented in a multi-state model.

**Supplementary Fig. 3.** Covariate balance using standardised mean differences at five day grace period before and after applying inverse probability of artificial censoring weighting


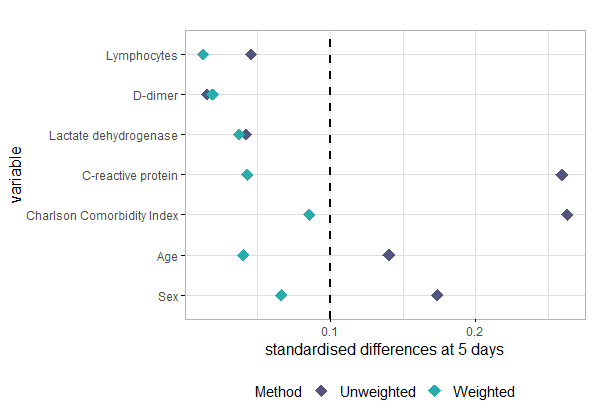


**Notes:** interpretation of the standardized differences: <0.1 adequate balance and effect sizes are considered to be very small; 0.1-0.25 not too alarming imbalance and effect sizes are considered to be small; >0.25 serious imbalance [1–3].

**Supplementary Fig. 4.** Covariate balance using standardised mean differences at five day grace period before and after applying inverse probability of artificial censoring weighting, complete dataset with missing value imputation, *n=599*


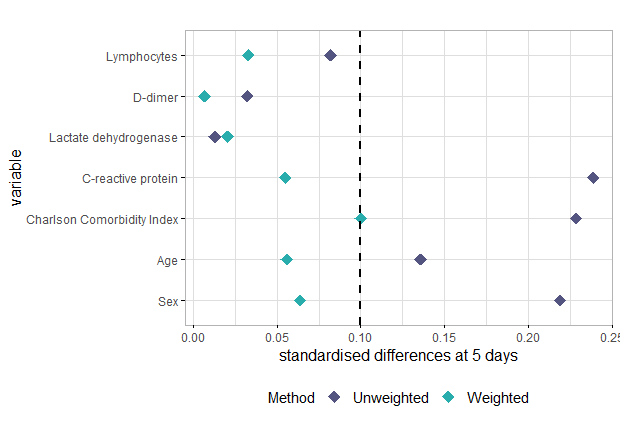


**Notes:** interpretation of the standardized differences: <0.1 adequate balance and effect sizes are considered to be very small; 0.1-0.25 not too alarming imbalance and effect sizes are considered to be small; >0.25 serious imbalance [1–3].

**Interpretation:** none of the standardized differences exceeded 0.1 or 10% after weighting. Thus, indicating that the patient’s characteristics were good balanced between the X-treated and non-X-treated arm after performing the weighting. In the weights, we included all the covariates that were included in the primary analysis: sex, age, the Charlson Comorbidity Index, oxygen saturation (%), as well as the inflammation markers C-reactive protein (mg/L), lactate dehydrogenase (U/L), D-dimer (ng/mL) and lymphocytes (×106/L).

**Supplementary Fig. 5.** Weighted estimated cause-specific cumulative hazards from the normal hospital ward using the Nelson-Aalen estimator, complete dataset with missing value imputation, *n=599*


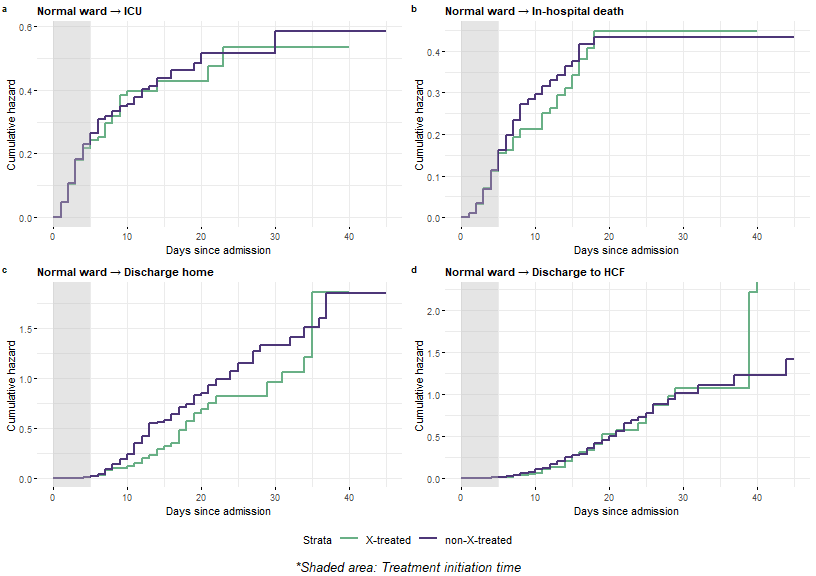


**Notes: a** transition from normal ward to ICU. **b** transition from normal ward to in-hospital death. **c** transition from normal ward to discharge home. **d** transition from normal ward to another healthcare facility (HCF).

**Interpretation:** the cumulative hazards between the two arms were similar during the follow-up period for all the outcomes.

**Supplementary Fig. 6.** Weighted results for transition rates starting from hospital admission, complete dataset with missing value imputation, *n=599*

**
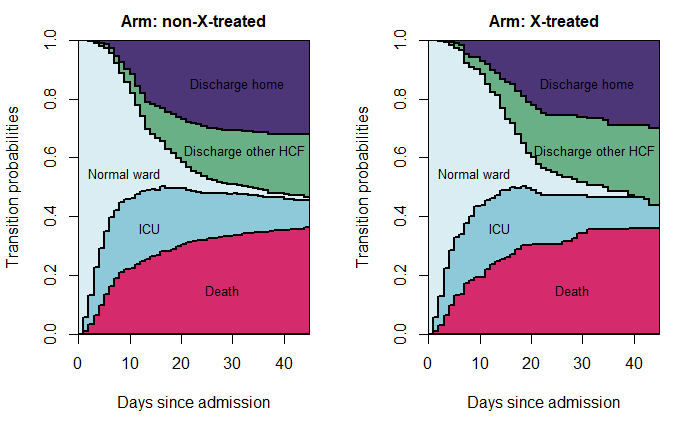
**

**Notes:** State 1: normal ward; State 2: admission to the intensive care unit (ICU); State 3: in-hospital death; State 4: discharge to home; State 5: discharge to another healthcare facility (HCF)

**Transition probabilities at 45 days**

- The transition rates of the outcomes we investigated were similar between the two treatment arms at 45 days of the follow-up (Supplementary Fig. 6).
- The probability of in-hospital death were similar for the both arms 36.1%.
- The probability of being discharged home were 30.1% for the X-treated arm and 32.2% the non-X-treated arm.
- The probability of being discharged to another health care facility were 25.9% for the X-treated arm and 21.3% the non-X-treated.

**References**

1. Stuart EA, Lee BK, Leacy FP. Prognostic score–based balance measures can be a useful diagnostic for propensity score methods in comparative effectiveness research. Journal of Clinical Epidemiology. 2013;66:S84-S90.el. https://doi:10.1016/j.jclinepi.2013.01.013.

2. Austin PC, Stuart EA. Moving towards best practice when using inverse probability of treatment weighting (IPTW) using the propensity score to estimate causal treatment effects in observational studies. Stat Med. 2015;34:3661–79. https://doi:10.1002/sim.6607.

3. Gupta S, Wang W, Hayek SS, Chan L, Mathews KS, Melamed ML, et al. Association Between Early Treatment With Tocilizumab and Mortality Among Critically Ill Patients With COVID-19. JAMA Intern Med. 2021;181:41–51. https://doi:10.1001/jamainternmed.2020.6252.
